# Correction: The green reduction of graphene oxide

**DOI:** 10.1039/d4ra90142h

**Published:** 2024-12-11

**Authors:** M. T. H. Aunkor, I. M. Mahbubul, R. Saidur, H. S. C. Metselaar

**Affiliations:** a Department of Mechanical Engineering, Faculty of Engineering, University of Malaya 50603 Kuala Lumpur Malaysia h.metselaar@um.edu.my +603 7967 5317 +603 7967 4451; b Center of Research Excellence in Renewable Energy (CoRE-RE), Research Institute, King Fahd University of Petroleum & Minerals (KFUPM) Dhahran 31261 Saudi Arabia mahbub_ipe@yahoo.com

## Abstract

Correction for ‘The green reduction of graphene oxide’ by M. T. H. Aunkor *et al.*, *RSC Adv.*, 2016, **6**, 27807–27828, https://doi.org/10.1039/C6RA03189G.

In this correction, we alert readers to a correction^1^ that has been published in relation to Fig. 8. Fig. 8 was originally published in *Carbon*.^2^

Readers are advised to refer to the corrected figure published in the correction notice.^1^

The Royal Society of Chemistry apologises for these errors and any consequent inconvenience to authors and readers.
